# Surface Enhanced Raman Spectroscopy of Lactoferrin Adsorbed on Silvered Porous Silicon Covered with Graphene

**DOI:** 10.3390/bios9010034

**Published:** 2019-02-28

**Authors:** Sergey Zavatski, Nadia Khinevich, Kseniya Girel, Sergey Redko, Nikolai Kovalchuk, Ivan Komissarov, Vladimir Lukashevich, Igor Semak, Kahramon Mamatkulov, Maria Vorobyeva, Grigory Arzumanyan, Hanna Bandarenka

**Affiliations:** 1 Laboratory of Applied Plasmonics, Belarusian State University of Informatics and Radioelectronics, 220013 Minsk, Belarus; sergeyzavatski13@gmail.com (S.Z.); khinevichnadia@gmail.com (N.K.); k.girel@bsuir.by (K.G.); 2 Laboratory of Materials and Structures of Nanoelectronics, Belarusian State University of Informatics and Radioelectronics, 220013 Minsk, Belarus; ml.redkov@gmail.com; 3 Laboratory of Integrated Micro- and Nanosystems, Belarusian State University of Informatics and Radioelectronics, 220013 Minsk, Belarus; n.kovalchuk@bsuir.by (N.K.); komissarov@yahoo.com (I.K.); 4 Laboratory of Nutrition and Sports Physiology, Institute of Physiology of the National Academy of Sciences of Belarus, 220072 Minsk, Belarus; lukashvs@rambler.ru; 5 Department of Biochemistry, Belarusian State University, 220030 Minsk, Belarus; semak@bsu.by; 6 Laboratory of Neutron Physics, Joint Institute for Nuclear Research, 141980 Dubna, Russia; hero170184@mail.ru (K.M.); vmu.chemist@mail.ru (M.V.); arzuman@jinr.ru (G.A.); 7 Dubna State University, 141982 Dubna, Russia

**Keywords:** surface enhanced Raman scattering, silver, porous silicon, graphene, lactoferrin, attomolar concentration

## Abstract

We registered surface enhanced Raman scattering (SERS) spectra of the human lactoferrin molecules adsorbed on a silvered porous silicon (*por*-Si) from 10^−6^–10^−18^ M solutions. It was found that the *por*-Si template causes a negative surface potential of silver particles and their chemical resistivity to oxidation. These properties provided to attract positively charged lactoferrin molecules and prevent their interaction with metallic particles upon 473 nm laser excitation. The SERS spectra of lactoferrin adsorbed from 10^−6^ M solution were rather weak but a decrease of the concentration to 10^−10^ M led to an enormous growth of the SERS signal. This effect took place as oligomers of lactoferrin were broken down to monomeric units while its concentration was reduced. Oligomers are too large for a uniform overlap with electromagnetic field from silver particles. They cannot provide an intensive SERS signal from the top part of the molecules in contrast to monomers that can be completely covered by the electromagnetic field. The SERS spectra of lactoferrin at the 10^−14^ and 10^−16^ M concentrations were less intensive and started to change due to increasing contribution from the laser burned molecules. To prevent overheating the analyte molecules on the silvered *por*-Si were protected with graphene, which allowed the detection of lactoferrin adsorbed from the 10^−18^ M solution.

## 1. Introduction

**Figure 1:**
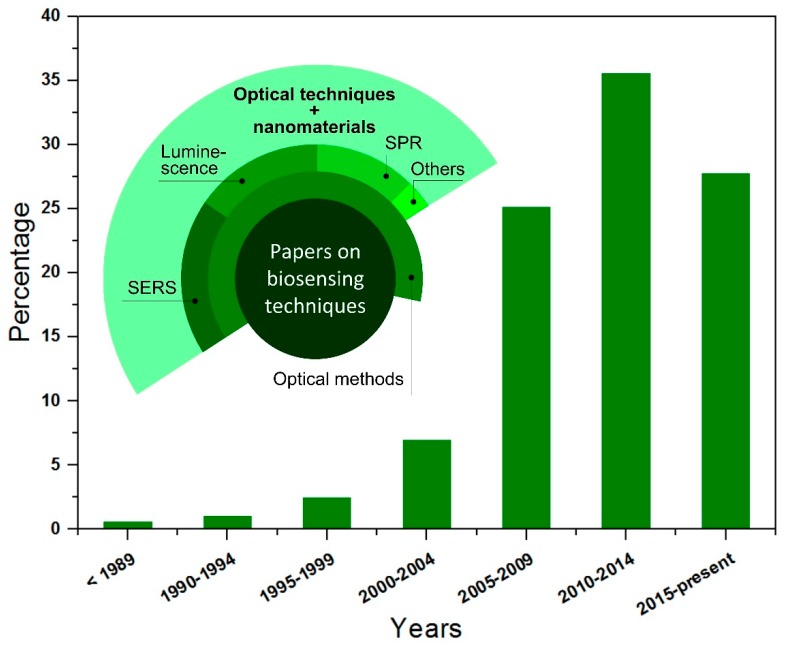
Number of papers on biosensing by year; inset shows the distribution of papers on biosensing based on the technique used (Google Academy, accessed on 30 November 2018).

The development of biosensing techniques to overcome the problem of reliable detection, identification, and structural study of diverse bioorganic molecules at ultralow concentrations is still an urgent objective of specialists in many spheres including medicine, biology, forensics, ecology, pharmaceutics and so on. This is proven by the statistics on “biosensing” papers, the number of which has increased greatly in the last years (Figure 1). Recently, there has been a rapid growth of interest in biosensing studies involving a variety of optical systems, devices and methods combined with nanomaterials, which are able to find molecules in physiological liquids and living cells at extremely low concentrations (Figure 1, inset) [1,2,3,4,5,6,7,8,9,10]. The main goals of these studies include non-invasive tests, medical diagnostics and therapy of different types of cancer [6,8], brain diseases [9], viruses-induced illnesses [10] and other dangers to human health. Accuracy and reliability are important requirements of desirable biosensing to reduce risks of false results. Raman spectroscopy has been considered an appropriate approach for biosensing [11]. Most chemical compounds have their own unique Raman spectrum signature, which allows identifying the molecule in the same way as one establishes a person’s identity by determining fingerprints. The undoubted advantages of Raman spectroscopy over other methods are its non-invasiveness, high resolution, negligible Raman intensity of water-based solvents, short term (a few minutes) analysis, non-complicated sample preparation, single excitation wavelength, etc. On the other hand, this technique has a major drawback that is a low sensitivity, which limits its practical application. The reason for this lack is that only one Raman photon out of 10^6^–10^8^ photons of incident light can be scattered. However, there is an effective way to solve this problem via the placement of analyte molecules on a surface of nanostructures of coinage metals that provides a great enhancement of the Raman signal by several orders of magnitude [12,13,14]. This effect is known as surface enhanced Raman scattering (SERS). The metallic nanomaterials are accepted to be called SERS-active substrates. The major contribution to the Raman signal enhancement is due to the surface plasmon resonance in metallic nanostructures arising under laser excitation [12,13,14]. At the same time, charge transfer between molecules of analyte and metallic nanostructures can also cause a signal increase [12,13,14]. Apart from its outstanding sensitivity, the Raman spectroscopy exploiting the SERS effect has additional benefits, such as the inhibition of luminescence of organic analytes by the metal particles and the selectivity provided by the proper functionalization of the SERS-active substrates. Therefore, the SERS-spectroscopy provides an opportunity of an ultrasensitive biochemical assay including precise identification and study of different molecules.

A special interest of SERS-spectroscopy has been paid to the detection and study of bioorganic macromolecules, in particular, to proteins that possess antiseptic properties, as they are prospective in the development of nanomaterials for the prevention and therapy of bacteria/virus-induced diseases [15]. Lactoferrin is a bright example of such proteins. It is a non-heme mammalian iron-binding glycoprotein, belonging to the transferrin family [16]. In addition to the iron transport, lactoferrin modulates immune responses, has antioxidant activity and anti-inflammatory properties, and participates in the regulation of cell growth and differentiation [17]. Lactoferrin is present at the highest concentration (1 mg/mL) in the milk of humans and other mammals [18]. It can also be found in a lower amount in blood plasma, neutrophils, saliva, bile, pancreatic secretion, and tears [19]. For example, the lactoferrin concentration in the tear of a healthy person varies between 10^−5^ and 2⋅10^−5^ M depending on the age [20]. When an eye is diseased, the lactoferrin concentration decreases more than the order of magnitude and its precise control in the tear liquid can help to distinguish causes of eye illness [21]. It was also revealed that lactoferrin affects cell growth (at 10^−8^–10^−6^ M) and sucrase and lactase mRNA expression (at 10^−10^ M) [22]. It is an urgent challenge to distinguish lactoferrin in a mixture of different proteins, when the SERS technique is used. Some features of the lactoferrin Raman spectra can be found in the literature [23] but they are rather subtle. At the same time, the lactoferrin detection in its water solutions is very important, as they can be used in the therapy of different diseases including cancer. Recently, it was reported that exogenous lactoferrin can affect the rate of tumor cells growth [24]. In addition, it was shown that recombinant human lactoferrin has a beneficial effect on the intestinal microflora, ultrastructure of the liver and intestines, activated lipid metabolism and steroidogenesis [25]. Proper therapy will require the precise control of this protein concentration in its solutions for oral, drip administration, etc. Therefore, monitoring the changes of the amount of this protein at submolar level in liquids is of great interest to research areas related to biomedicine.

It should be noted that nowadays an enzyme-linked immunoassay (ELISA) and a high performance liquid chromatography (HPLC) are widespread techniques for the basic research and clinical investigation of proteins. Conducting the ELISA includes at least one antibody with a specific immune response against the antigen of interest. The ELISA detection methodologies have a high specificity, provide enough reproducibility of data and allow the quantitative investigation of analytes [26]. However, the general shortages of the conventional ELISA are that it is a tedious/laborious procedure, has limited multiplexing options, and operates with relatively large sample volumes. Moreover, the detection limit of the conventional ELISA is near a nanomolar concentration, which is inadequate to reach the clinical threshold of many proteins’ biomarkers, especially in the early stage of diseases [27]. For example, Ying Pan and co-authors [28] reached the 0.145 ng/mL detection limit using the ELISA technique. In Reference [29] the detection limit of the lactoferrin of 2.5 ng/mL was reported. In addition, the ELISA is known to take 24 h or even several days to get results. Thus, there is an urgent need to find a faster analysis. Liquid chromatography (LC) is a physical separation technique conducted in the liquid phase. The HPLC approach is a modern form of the LC method that uses small-particle columns, through which the mobile phase is pumped at a high pressure. The HPLC has advantages over other methods because it is capable of the multicomponent analysis of real-life samples and complex mixtures with good resolution and sensitivity. This method has been employed for determining all major whey proteins including lactoferrin. However, the HPLC suffers from several well-known disadvantages. First, there is no universal detector, hence the detection is more problematic if the analyte does not absorb ultraviolet (UV) or cannot be easily ionized for mass spectrometric detection. Second, the HPLC has many operating parameters and is more inconvenient for routine measurements. Third, although the HPLC can often simplify and speed up the solution separation process, the cost of the HPLC equipment can become tremendous. Finally, similar to the ELISA technique, the HPLC hardly provides a suitable detection limit of protein biomarkers for clinical application. For example, Zhang et al. achieved the detection limit of 0.6 mg/100 g for the lactoferrin [30]. Krol et al. showed the 8.7 mg/L detection limit for the lactoferrin standard during calibration of the chromatographic system [31]. Compared with these techniques, the SERS-spectroscopy exhibits a unique blend of advantages, such as very high sensitivity, non-invasive probing, compatibility with water solutions, simplicity of a sample preparation, label-free monitoring for specific analyte in complex mixtures [32], and a small sample volume. Moreover, SERS-spectroscopy does not require specific and expensive equipment, which allows routine measurements to be conducted even by a non-specialist.

Despite SERS-spectroscopy allows one to study molecules at extremely low concentrations, studies devoted to the deep investigation of lactoferrin by this method have not been presented yet. This is probably connected with difficulties that appear during the SERS analysis of organic species of high molecular weight. In general, the enhancement of Raman signals provided by metallic nanostructures depends on the substrate nanotexture, the type of metal, the excitation wavelength, its polarization, the orientation of the bonds, which vibrations we have to detect, the possible anharmonicity of oscillations during adsorption on a substrate, chemical bonds with the substrate, etc. These conditions greatly influence the results of SERS analysis, especially during the study of large molecules such as DNAs, proteins, and even peptides [11,33]. Their conformation is changed on the solid substrate. Some bonds cannot be located in the “hot spots”, resulting in a weak reproducibility of the SERS spectra [34]. Difficulties have been also observed at low concentrations, when there is a need of an extremely high enhancement factor to detect substance. They are because of irreversible changes of the analyte molecules due to their interaction with metallic nanostructures and destruction upon the photothermal effect from the laser [35]. What is more, silver nanoparticles, which are very attractive for SERS-spectroscopy as they provide the greatest activity referred to electromagnetic mechanism, show the surface plasmon resonance in a blue range close to UV. The combination of d-metal and UV excitation can also result in organic denaturation. In addition, silver nanostructures are known to be positively charged. This is a benefit if there is a need to penetrate via the cell membrane of harmful species or to interact with protein for a creation of antiseptic nanoparticles [15,36]. However, it turns into a disadvantage for SERS-spectroscopy because silver nanoparticles are easily oxidized under ambient conditions. Thus, they show a losing reproducibility and weakening intensity of the SERS signal in the course of time.

The present work pursued the goal of overcoming the above-mentioned hurdles of the SERS spectroscopy of biomolecules with a high molecular weight that include: (i) The weak reproducibility of the SERS spectra due to partial location of the molecule in the hot spot and (ii) destructive effect from the laser-metal particle interaction. We also aimed at finding the minimal concentration of the test macromolecules in the water solutions, at which an analyte can be detected. The recombinant human lactoferrin was used as test analyte molecules. To achieve these objectives, we proposed to study and use the beneficial cooperative effect from the SERS-active substrates based on a silvered *por*-Si and the protection of the analyte molecules with graphene. The silvered *por-*Si has been already successfully utilized as the SERS-active substrate [37,38,39,40] demonstrating an exceptional detection limit down to the femtomolar concentration [41,42,43,44], a high SERS signal reproducibility [45,46,47] and a long shelf life of up to two years [47], which is not typical for the silver-based SERS-active substrates. Despite the last feature, which has not been plausibly explained before, we suppose that the silver nanostructures on the *por*-Si surface have an improved chemical resistance preventing their oxidation and agglomeration. They must be strongly anchored on the negatively charged *por*-Si surface, which is composed of a huge number of nanocracks and roughness with broken bonds [48]. Therefore, the silver nanostructures on the *por*-Si are expected to show less affinity to the oxidation and aggressiveness to organic molecules. At the same time, graphene, which is known to enhance the Raman signal itself [49], will contribute to the amplification of the Raman signal being placed on the surface of metal nanostructures [50,51]. What is more, if graphene covers the analyte molecules, it can protect them from the environment and provide a heat sink due to the high thermal conductivity. This will prevent the photo- and thermal-induced destruction of analyte.

## 2. Materials and Methods

Monocrystalline n^+^-type Si wafers (*c*-Si) were used for the fabrication of the *por*-Si template. Prior to the *por*-Si formation, the Si wafers were cleaned in a solution of NH_4_OH, H_2_O_2_ and H_2_O mixed at a volume ratio of 1:1:4. Native SiO_2_ was removed from the Si wafer in diluted HF (4.5%). The *por*-Si layers of a 5 μm thickness, a 75% porosity and a 70–100 nm pore diameter were fabricated by an electrochemical anodization according to the procedure described elsewhere [52]. The electrochemical anodization process was carried out with the potentiostat/galvanostat AUTOLAB PGSTAT302n. After anodization, a silver immersion deposition on the *por*-Si was performed from a water-ethanol solution of 3 mM AgNO_3_ for 70 min resulting in a dominant deposition of silver nanoparticles on an external surface of the porous material [53]. Then the samples were rinsed with deionized water and air-dried. The photo of the resulting substrate is shown in Figure S1. The silvered *c*-Si samples were also prepared using the same deposition conditions to compare their surface potential with that of the silvered *por*-Si. 

To measure the surface potential of the silvered *c*-Si and *por*-Si, a platinum reference electrode was used. The electrode was located near the sample surface. The surface potential measurements of the samples were carried out in deionized water and 10^−6^ M lactoferrin solution.

Chemical vapor deposition (CVD) was used for the synthesis of graphene. Electrochemically polished copper foil was annealed for 1 h at 1050 °C in Ar and H_2_ gases’ flow with a rate of 160 and 150 cm^3^/min, respectively. Then a 4 cm^3^/min methane flow was introduced into the CVD reactor for 10 min. We used a wet-chemical transfer of graphene without a polymer support. A preparation of the graphene films for the transfer was carried out as follows: (i) The underside of the copper foil was processed for 3 min in a solution of HNO_3_:H_2_O, mixed in a volume ratio of 1:3 and (ii) the copper foil was completely dissolved in an aqueous solution of FeCl_3_. As a result, the graphene film remained floating on the surface of the solution. Before the graphene transfer, this solution was carefully replaced several times with distilled water, which facilitated its purification from the products of chemical reactions. This operation was carried out with the aim of minimizing undesirable adsorption of various compounds on the surface of the SERS-active substrate, which could distort the results of subsequent measurements. When working on the transfer process, the substrate both containing the *c*-Si and silvered *por*-Si areas was immersed in the solution, placed under the graphene film and lifted up from the solution. In this case, the graphene film remained on the surface of the substrate.

A morphology of the experimental samples was studied with the scanning electron microscope (SEM) Hitachi S4800, which provided 1 nm resolution. The elemental composition of the samples was determined using SEM Cambridge Instruments Stereoscan-360 with a Link Analytical AN 10,000-energy dispersive X-ray (EDX) analyzer. The diameter of the focused electron beam was about 2 μm. The depth of analysis did not exceed 1 µm.

The simulations of electric field strength and heat transfer in the silvered *por*-Si, both free of graphene and covered with it, were performed using COMSOL Multiphysics 5.3a.

Organic dye Rhodamine 6G (R6G) and recombinant human lactoferrin [24,25] were used as analytes for the SERS measurements. The R6G dye (purchased from Sigma–Aldrich) and lactoferrin (received from the Department of Biochemistry of the Belarusian State University) had a 98% purity. Recombinant human lactoferrin from milk of transgenic goats was purified using cation-exchange chromatography according to the procedure described elsewhere [24,25]. The physicochemical characteristics of the recombinant lactoferrin were similar to those of natural human lactoferrin (from woman’s milk) as revealed by mass spectrometry and peptide mapping [24,25]. According to Reference [54], human milk lactoferrin is saturated with iron up to 10%. R6G was chosen to study the influence of graphene on the SERS-activity of the silvered substrates. The analytes’ powders were dissolved in a deionized water to obtain the solutions of 10^−6^–10^−18^ M concentrations. The solutions of lactoferrin were clear and soluble at different concentrations. Good solubility of lactoferrin in water solutions is typical for this protein even while the pH changes [55]. The *c*-Si and silvered *por*-Si samples of a 0.25 cm^2^ surface area were immersed in 1 mL of the analytes’ solutions for 1 h before the measurements and then rinsed in deionized water for 30 s to remove an excess of the analyte molecules. The lactoferrin retained its solubility during the immersion of the silvered samples in its solutions. The SERS spectra were registered with a 3D scanning confocal microscope Confotec NR500. Laser with a 473 nm wavelength and a spot diameter about 600 nm was used to excite the samples. Laser had a 25 mW power that decreased via optical system and ×100 objective down to 1.45 mW. We used both single point and mapping SERS measurements. They are described in more detail below, in the paragraphs related to the SERS-spectroscopy of different samples. To check the repeatability of the results of the spectroscopic study, we recorded the SERS spectra in two areas of the single substrate, one area of the other substrate from the same batch and one area of the substrate from a different batch for each research stage.

## 3. Results and Discussion

### 3.1. Characterization of SERS-Active Substrates

The fabrication technique of the SERS-active substrates used in this work has been already reported [47]. It is known that particles of the coinage metals (including silver) can be deposited on the *por*-Si surface because of oxidation of silicon atoms with silver ions from the solution and their following reduction in an atomic form [38,40,56]. In contrast to *c*-Si, the *por*-Si material provides more electrons for reduction and centers for nucleation of the silver atoms due to an extremely developed nanostructured surface. In this work, we used the silvered *por*-Si substrates that have been well-studied before [53,57]. The SERS-active layer of these substrates presents non-continuous film composed of quasi-spherical silver particles of polycrystalline nature, which dominating sizes are in the range of 10–150 nm, however, some of them have diameters of 150–700 nm (see Figure S2). Namely, these structures were found to demonstrate the greatest shelf life [47]. To check the idea of a negative surface charge of the silvered *por*-Si, which prevents its oxidation, we compared the surface potential of the *c*-Si and *por*-Si samples in water. It was found to be equal to +0.01 V for the silvered surface of the flat silicon and to −0.3 V for that of *por*-Si. Next, we studied the elemental composition of the as-prepared substrates and the substrates air-aged for six months. All the EDX spectra looked rather similar and it was possible to reveal the content of the samples via their quantitative analysis. That is why we presented only one EDX spectrum typical for the silvered Si-based samples on Figure S3. An amount of oxygen in the silvered *c*-Si increased almost twice while the sample based on the *por*-Si showed a nearly constant amount of oxygen (Table 1). The increase of the oxygen amount cannot be explained by the silicon oxidation in the air. The silver deposition from the water-based solutions leads to the formation of the silicon dioxide layer that protects the underlying *c*-Si substrate from the further oxidation. Therefore, the silver particles deposited on the *por*-Si are characterized by a better stability due to the resistance to oxidation. It should be considered that the higher oxygen amount in the silvered *por*-Si (in contrast to that based on *c*-Si) is due to the greater surface area of the porous material for the layer of the 1 μm thickness analyzed by the EDX spectrometry. The other beneficial effect is the negative charge of the silver particles provided by the *por*-Si template. This will very likely prevent interactions with the bioorganic analyte that is typical for positively charged silver particles [15,50]. As a result, the SERS-spectra will show objective information on the structure of the studied biomolecules.

### 3.2. Characterization of Graphene-Containing Films

**Figure 2:**
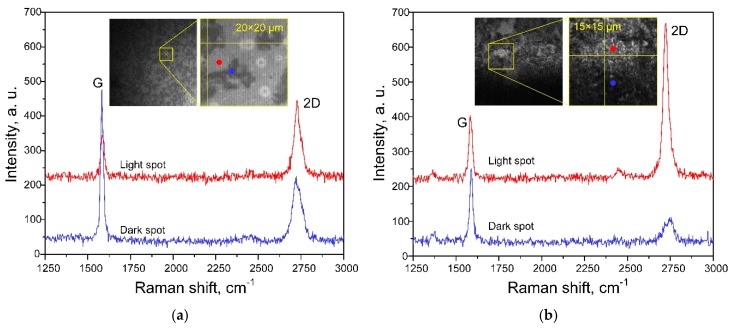
(**a**) Raman and (**b**) SERS spectra of graphene-containing film on the (**a**) *c*-Si and (**b**) silvered *por*-Si substrates registered in the light and dark spots. Insets show the Rayleigh images of the graphene-containing film.

It is known that graphite’s Raman spectra can contain three intensive bands—G, 2D, and D [58]. The G band (1582 cm^−1^) is associated with a doubly degenerated phonon mode of E2g symmetry from the center of the Brillouin zone. The 2D band (around 2710 cm^−1^) is due to resonant light scattering with the participation of two phonons of the same energy, but with the opposite direction of the pulse, and gives information about the ordering of graphite (graphene) layers. The band D (1352 cm^−1^) characterizes defects in the graphite (graphene) structure. To characterize a film formed by the CVD method we first made Rayleigh images of graphene-containing film transferred to the *c*-Si surface and measured its Raman spectra (Figure 2a). Following the Rayleigh images, dark and light spots were observed in the film, which are supposedly caused by irregularity of the graphene-containing coating. The Raman spectra were registered in both spots. The 2D band positions were shifted to the higher frequencies. This shift can be caused by mechanical stress in the film. Paper [59] reports that the number of graphene layers is calculated from a ratio of intensities of G and 2D bands. I_G_/I_2D_ ratio increases nearly linearly in the range of 0.24–0.75 from single to eight layers of graphene [60]. Analysis of the Raman spectra in the light spots showed that they are only composed of 1–8 layers of graphene whereas the dark spots present a highly oriented pyrolytic graphite (HOPG).

The same assay was performed for the samples of the silvered *por*-Si (Figure 2b). One can see that the silvered *por*-Si provides twice the increase of the Raman signal due to the SERS-activity. To find a number of the graphene layers we made a quantitative analysis of the Raman and SERS spectra (see Table S1). The graphene monolayer can be found on both substrates. This follows from the position of the G band (1587 cm^−1^), the full width at half maximum (FWHM) of the 2D band (29 cm^−1^) and the I_G_/I_2D_ ratio. The band associated with deformation, disorder, and defects in crystalline structure of graphene (~1350 cm^−1^) cannot be found in the spectra evidencing its good quality [59].

### 3.3. SERS-Activity of the Silvered por-Si Covered with Graphene

**Figure 3:**
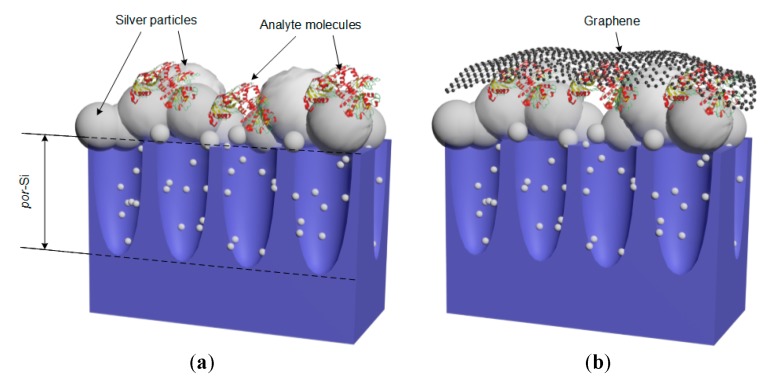
Schematic views of the SERS-active substrates: (**a**) The silvered *por*-Si with adsorbed analyte molecules; (**b**) the silvered *por*-Si with adsorbed analyte molecules coated with graphene.

**Figure 4:**
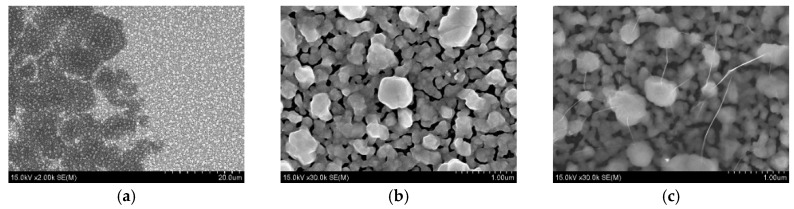
SEM top view images of the silvered *por*-Si: (**a**) Interface between graphene-covered and graphene-free areas at low magnification; (**b**) graphene-free surface; (**c**) graphene-covered surface.

In this section, we experimentally studied the effect of graphene on the SERS-spectra of the test analyte R6G. First, the SERS-spectrum of the analyte on the silvered *por*-Si was measured. Next, the sample with analyte was covered with graphene-containing film and the SERS-spectra in the light and dark points were registered. Schematic representations of the SERS-active substrates are shown on Figure 3. Figure 4 demonstrates that the morphology of the SERS-active substrates was not significantly changed after coating with graphene.

**Figure 5:**
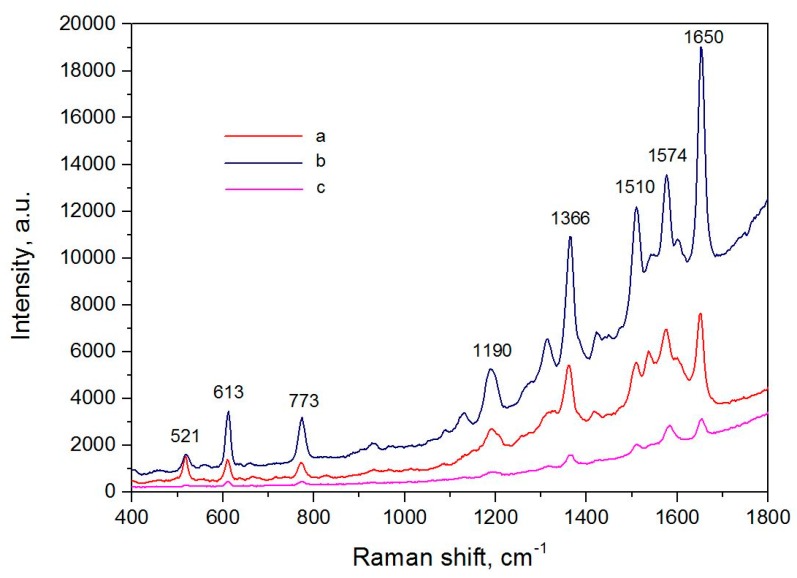
SERS-spectra of R6G molecules adsorbed from 10^−6^ M solution on the silvered *por*-Si: (**a**) Free of graphene; (**b**) covered with graphene, in the light spot; (**c**) covered with graphene, in the dark spot.

Figure 5 shows SERS spectra of the R6G molecules adsorbed on the silvered *por*-Si free of graphene and covered with it. Each spectrum was measured at the laser power reduced by one order of magnitude. Laser exposition was 2 s. A deviation of the SERS intensity did not exceed 7% for all the samples. It can be seen that the intensity of the SERS spectrum of R6G adsorbed on the silvered *por*-Si under the graphene-containing film in the light spot is 2–2.5 times more than that for the analyte molecules, which are not protected with the carbon nanostructure. At the same time, the SERS intensity of the analyte spectrum in the dark spot under graphene is significantly weaker than that of the substrate free of graphene. The R6G bands at 1310, 1510, and 1574 cm^−1^ are suppressed in the SERS-spectrum registered on the graphene-free substrate and partially overlapped by the bands associated with amorphous carbon (1339 and 1597 cm^−1^) [61], i.e., the analyte burned during the measurements whereas the graphene protection eliminated this effect. Therefore, the film containing 1–8 graphene layers provides an additional enhancement of the SERS signal and prevents analyte destruction. Contrariwise, the HOPG spots inhibit the SERS-activity but also protects the analyte from the burning.

### 3.4. Simulation of the Silvered por-Si Covered with Graphene

A better understanding of the graphene effect on the SERS measurements requires computer simulations of an electric field and a temperature distribution in the silvered *por*-Si free of graphene and covered with it. Silver hemispheres with a diameter of 80 nm (an average size of the silver particles in the real structure estimated from the SEM images) were placed on a thick substrate at a distance between their centers of 85 nm. Refractive index of the substrate was chosen to correspond with that of an amorphous silicon. We used thermal properties of a slightly oxidized mesoporous silicon (thermal conductivity 34 W/(m⋅K)) for the substrate in heat transfer calculations. The graphene layer was optically and thermally thin (three monolayers) with a 2500 W/(m⋅K) thermal conductivity.

The laser heating of the structure was simulated in two steps. First, we solved a 2D frequency-domain wave equation for the electric field with a finite element method in order to obtain the electromagnetic losses from the electromagnetic waves as a heat source. To simulate the excitation laser light, we used a Gaussian electromagnetic wave in the scattered-field formulation. The beam propagated in the negative y-direction. The wavelength was 473 nm and the spot radius of the tightly focused beam was 305 nm. The incident power of the beam was 1 mW. Next, we solved a heat transfer in a solids stationary equation to see the equilibrium temperature distribution in our structure with the graphene layer and free of it.

The graphene-containing film was found to have no effect on the electric field strength between the silver particles, which is maximal in the center of the laser spot. The electric field distribution around the silver hemispheres typical for both graphene-free and graphene-covered substrates is presented in Figure S4. Simulations revealed that the maximum temperature is about 10 degrees lower in the graphene-covered structure (341 K vs. 350 K) (Figure S5a,b). Isothermal contours also showed a better heat propagation in a lateral direction in this structure (Figure S5c,d). The value of 350 K is very close to the point of lactoferrin denaturation [62] and laser heating can destruct protein’s molecules, especially at trace amounts. A decrease of the temperature provided by covering the SERS-active substrate with graphene provides the opportunity for the protection of the lactoferrin molecules from partial denaturation.

### 3.5. SERS-Spectroscopy of Lactoferrin Molecules

The initial SERS-measurement of lactoferrin was devoted to reveal if the silvered *por*-Si substrate provides the registration of its spectrum free of changes caused by the interaction of the bioorganic molecule with metallic particles and denaturation under laser irradiation. We suppose that lactoferrin is adsorbed nearly uniformly by the surface of the silvered *por*-Si because its isoelectric point is 8.3 while the measured pH of lactoferrin solutions at all concentrations was 6.5–7. Upon these conditions, the lactoferrin molecules are positively charged and attracted by the negatively charged silver surface. We measured the surface potential of the silvered *por*-Si during immersion into the water solution of lactoferrin and found that it increased from −0.3 to −0.2 V for 2 min. After that, the surface potential periodically deviated around the latter value by ±0.04 V. We suppose this deviation is connected with the surface charge changes. It shows that positive lactoferrin molecules are attracted and adsorbed by the negative metallic surface and as the potential increases, they are repulsed and slightly desorbed into the solution.

**Figure 6:**
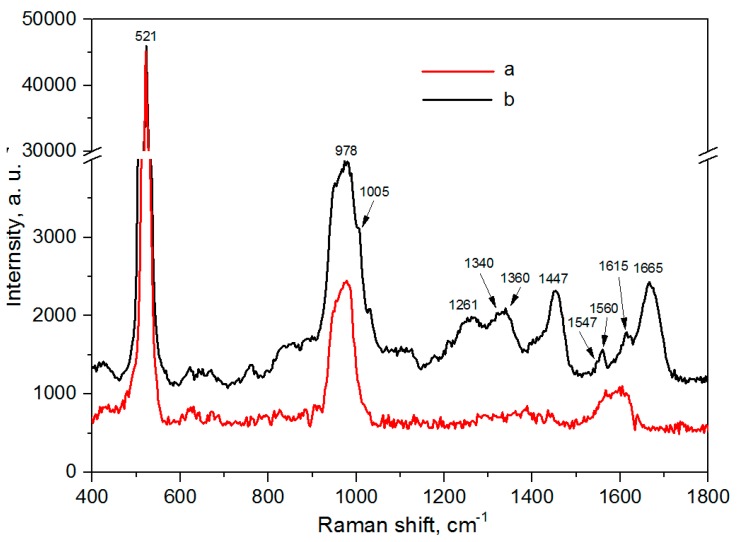
SERS spectra of the silvered *por*-Si: (**a**) Virgin; (**b**) after adsorption of the lactoferrin molecules from the 10^−6^ M solution.

Figure 6 shows the SERS spectra of the silvered *por*-Si and the lactoferrin molecules adsorbed on this substrate from 10^−6^ M solution. We aligned the spectra according to the position of the Si band at 521 cm^−1^. The SERS spectra were registered during 1 s at laser power 1.45 mW. The substrate’s spectrum has just bands signifying the *c*-Si (521 cm^−1^) and SiO_2_ (978 cm^−1^) contents. There is no evidence that silver oxide is in the sample, which proves the results described in Section 3.1. The SERS spectrum of lactoferrin is characterized by the presence of all bands typical for this analyte such as 1005 cm^−1^ (Phe), 1230–1300 cm^−1^ (Amide III and/or Tyr), 1340 and 1360 cm^−1^ (Trp), 1447 cm^−1^ (δCH_2_), 1547 and 1560 cm^−1^ (Trp), 1600–1615 cm^−1^ (Tyr) and 1665 cm^−1^ (Amide I) [11,63]. To show the similarity of the SERS spectra of lactoferrin, its Raman spectrum registered for the 10^−4^ M solution on the silicon can be found in Figure S6. A correlation of intensities of the bands in 1260–1290 cm^−1^ shows the hydrophobicity of the molecular environment of Trp residues. The bands between 1547 and 1560 cm^−1^ show the orientation in the Trp indole ring in relation to the peptide backbone, doublet signifies about two different orientations. Considering the Amide I band, we needed to analyze its position which can vary from 1620 to 1700 cm^−1^. In our case, it was at 1665 cm^−1^ and corresponds to the β-sheet content. It is known that lactoferrin contains both alpha helices and beta structure [62,64]. In [62] the authors estimated the percentage of alpha helices and beta strands from the Raman spectra during temperature changes. Heating the lactoferrin molecules caused a prevalence of residues in the beta structure but its percentage was found not to reach 100%. In our work, the heating effect from the laser can lead to a more prominent Amide I band, which position is typical for the beta structure. Thus, the less intensive Amide I band related to alpha helices will be overlapped with that of the beta strands. Here, it is very likely caused by the thermal effect from the laser. The other marker of the secondary structure is the Amide III band located between 1230 and 1300 cm^−1^ but here it can be hidden in rather intensive Tyr bands (1260–1290 cm^−1^).

**Figure 7:**
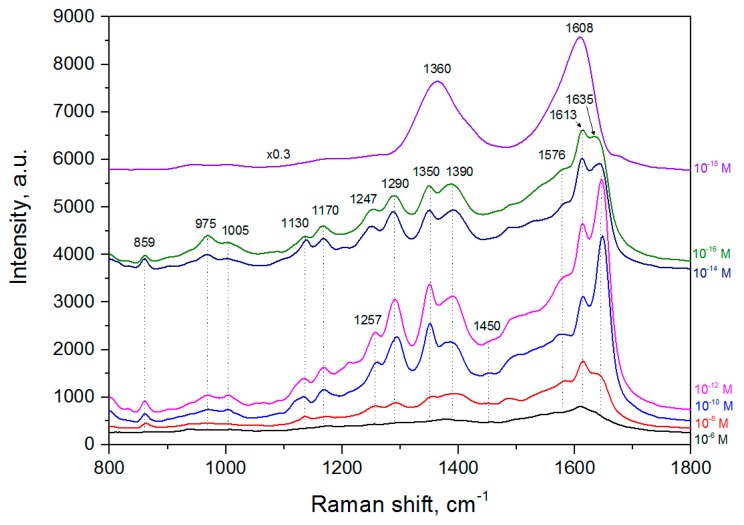
SERS spectra of the lactoferrin molecules adsorbed on the silvered *por*-Si.

Next, we revealed the minimal concentration of the lactoferrin solution at which its molecules are still detected after adsorption on the silvered *por*-Si. Figure 7 presents the SERS spectra of lactoferrin at 10^−6^ to 10^−18^ M concentrations, which were obtained by the collection and integration of the SERS spectra maps. We used two SERS-active substrates from different batches and scanned a 10 × 10 μm surface area of both substrates with a step of 1 μm. Each point of the maps was excited for 2 s. The laser power was decreased to 0.14 mW in contrast to the previous measurement to eliminate a harmful photothermal effect.

The SERS spectrum of lactoferrin adsorbed from 10^−6^ M is rather weak but the decrease of the concentration down to 10^−12^ M led to the enormous growth of the SERS signal. Lactoferrin mostly exists in tetramer and monomer forms [65,66]. Supposedly, tetramers are at concentrations higher than 10^−10^ M while the lower amount of this analyte leads it to turning into a monomer form. Tetramers are too large for the uniform interaction with the electromagnetic field from the silver particles to provide an intensive SERS signal from the top part of the molecules. At the same time, monomers can lay between the metallic structures and be completely excited. The SERS spectra at the 10^−14^ and 10^−16^ M concentrations have lower intensities and at 10^−18^ M the pattern typical for amorphous carbon is observed.

All the informative SERS spectra are characterized by the presence of this analyte bands. Some burning of the analyte with the concentration decrease is observed as bands related to the amorphous carbon (around 1339 and 1597 cm^−1^) become broader. We still observe bands at 1002–1005 cm^−1^ (Phe ring breathing mode), 1130 cm^−1^ (corresponding to C-N bonds), 1350 and 1380–1390 cm^−1^ (Trp residues), 1450 cm^−1^ (δCH_2_), 1610–1615 cm^−1^ (Tyr) and 1647–1650 cm^−1^ (Amide I). The bands at 859 cm^−1^ (the asymmetric C-C-S stretching of Met side) and at 1170 cm^−1^ (the CO bending of the phenolate ligand) are now prominent in contrast to the previous single measurement due to a better enhancement and spectra integration. We also see intensive Tyr bands at 1257–1260 and 1290 cm^−1^. Alternatively, one of these bands can be Amide III and signify the domination of the α-helical structure, which is also proved by the position of the Amide I band. In this case, the Amide I band related to the beta strands is hidden in more intensive bands of alpha helices. Therefore, the signature of only β-sheet structure in the spectrum registered for the higher laser power could be caused by overheating. Unfortunately, the bands at 1547 and 1560 cm^−1^ can be overlapped by the wide band of amorphous carbon with maxima at 1576 cm^−1^. However, the spectra analysis gives an idea that this doublet coalesced in one band shifted to 1560 cm^−1^ because no hint of any band at 1547 cm^−1^ can be found. If so, this can confirm that the prevalent orientation of the indole ring is at an intermediate angle.

**Figure 8:**
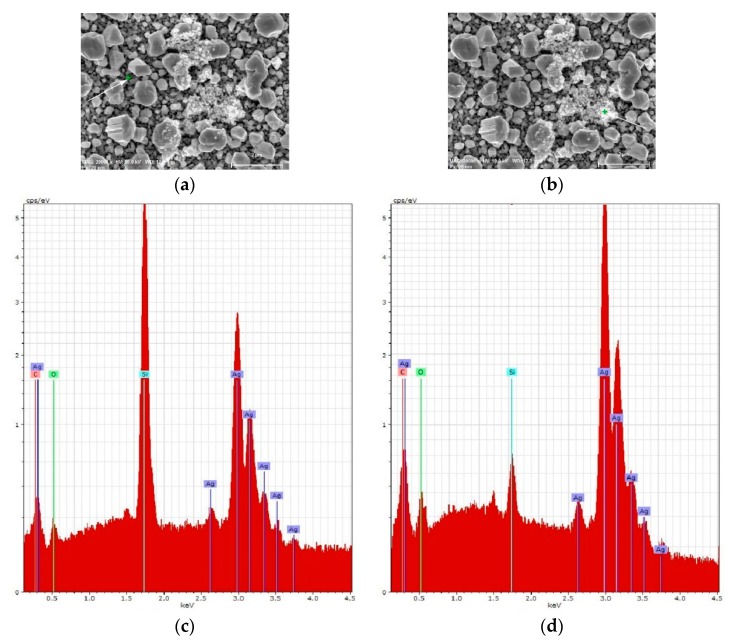
(**a**,**b**) SEM top view images of the silvered *por*-Si after adsorption of the lactoferrin molecules; (**c**,**d**) EDX spectra corresponded to the spots marked with a green cross on the SEM images.

**Figure 9:**
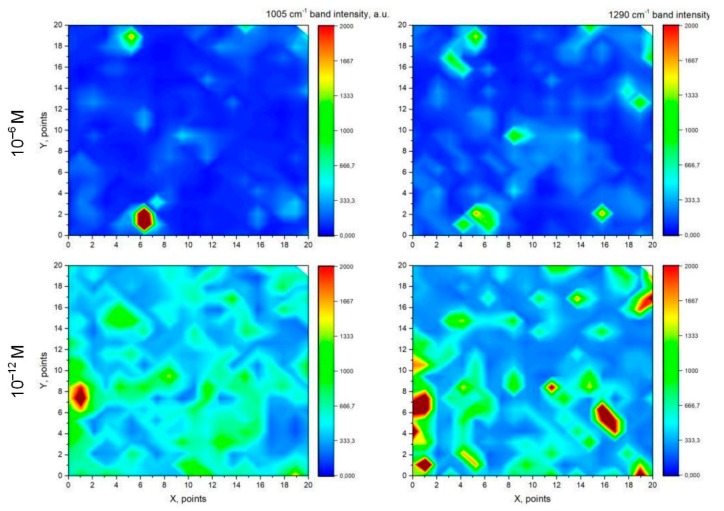
Distribution of the SERS-signal intensity at the 1005 and 1290 cm^−1^ bands in the SERS-spectra of the lactoferrin molecules adsorbed on the silvered *por*-Si from the solutions at 10^−6^ and 10^−12^ M. The size of each map is 10 × 10 μm.

For the deeper understanding of the adsorption of the lactoferrin molecules on the SERS-active surface they were studied by SEM and EDX after immersion to the lactoferrin solutions at different concentrations. It was revealed that at the 10^−6^ M and 10^−8^ M concentrations the surface of the samples was randomly covered by separate accumulations of nanoparticles with diameters varying from a couple to hundred nanometers (Figure 8a,b). The number of accumulations was about 10 per 1200 μm^2^ for both concentrations. However, an average area taken by the single accumulation was different: the average area of one accumulation is nearly 15 μm^2^ for the 10^−6^ M concentration and is ~7.5 μm^2^ for the 10^−8^ M concentration. Thus, the number of nanoparticles decreased with a lowering concentration. EDX analysis showed that the atomic percentage of carbon in these accumulations is more than that on the surface free of the accumulations (Figure 8c,d). Supposedly, these accumulations can be a result of the lactoferrin precipitation when its molecules are adsorbed on the SERS-active substrates. SEM views of the samples related to 10^−10^ M and less are not presented as they showed no difference with those free of lactoferrin. However, we were able to register the lactoferrin’s SERS spectra, i.e., its molecules were adsorbed on the substrate. Smaller nanoparticles containing the lactoferrin molecules are not observed on the SEM pictures but this can be caused by limitations in resolution. The volume of the lactoferrin monomer is 0.132 nm^3^ [67] and we can estimate its average diameter of ~0.65 nm. Considering the SEM resolution of 1 nm, it can be assumed that lactoferrin is adsorbed as monomer at concentrations lower than 10^−10^ M. To reveal the character of the lactoferrin molecules distribution we registered the SERS maps of the 10 × 10 μm area with a 500 nm step. The maps of the SERS intensity of the main protein bands were created. Two of them (according to the intensity of 1005 and 1290 cm^−1^ bands) for 10^−6^ and 10^−12^ M concentrations are presented in Figure 9. The sample with lactoferrin at the micromolar concentration was studied in the area of its molecules’ accumulation, which was found at the Rayleigh’s mode. The intensive SERS signal was not observed in the thick layer of accumulation as the electromagnetic field from the metallic particles is not able to excite them. In contrast, it was registered in the particular spots close to the accumulation, where the lactoferrin molecules in a lower amount were adsorbed. The case of picomolar concentration shows more points of the SERS-spectra of lactoferrin. The above-described results show that the lactoferrin molecules were adsorbed on the SERS-active substrate non-uniformly. The lactoferrin solutions with concentrations higher than 10^−10^ M led to the accumulation of the lactoferrin molecules in the nanoparticles (precipitations) with diameters of a couple to a hundred nanometers. The use of the more diluted solutions resulted in the accumulations size smaller than two nanometers or no accumulations at all.

We were able to analyze the lactroferrin molecules adsorbed on the silvered *por*-Si down to the 10^−16^ M concentration. However, at the 10^−18^ M concentration we found just the bands corresponded to the amorphous carbon. We suppose that the 10^−16^ M concentration is still enough to provide adsorption a plenty of molecules at several close to each other silver particles, which are in the laser spot. This allows getting the enhanced signal from a number of molecules located around the central point in the laser spot that is the most heated. Lower concentration leads to less amount of the adsorbed molecules, which cannot provide getting of proper enhancement within the laser spot, while burned species result in the high interference from vibration of amorphous carbon bonds.

**Figure 10:**
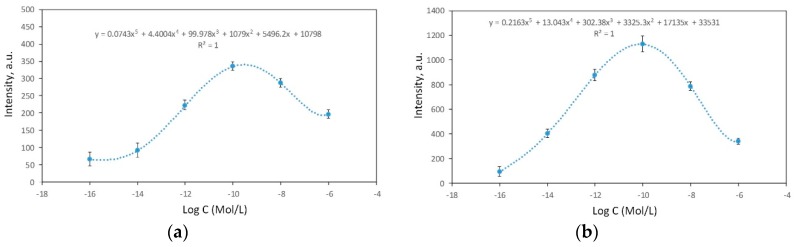
The calibration curves of the SERS intensities at the (**a**) 1005 and (**b**) 1290 cm^−1^ bands vs. the lactoferrin concentration. The curves were calculated using a polynomial fitting of the fifth order.

Usually an analyte concentration during the SERS measurements is estimated using a comparison of intensities of the selected bands reflected in a calibration curve. As a rule, the intensity of the signal decreases with the concentration lowering. This dependence results in a linear calibration curve. The data obtained in our work do not have an ordinary view, i.e., the SERS signal intensity of the analyte is not directly correlated with its concentration. This is caused by the specificity of adsorption and the distribution of the lactoferrin molecules and their structural form at different concentrations. Therefore, the calibration curves have a non-linear character. The intensities of the bands at 1005 and 1290 cm^−1^ were used (Figure 10) to find calibration curves that visualize the dependence of the SERS intensity on the lactoferrin concentration. The values of the SERS intensities for some concentrations overlapped, if the single band was selected. For instance, the SERS intensities of the 1005 cm^−1^ band were, very close in the pairs at the 10^−6^ M/10^−12^ M and 10^−14^ M/10^−16^ M concentrations. However, for the 1290 cm^−1^ band the other pairs were characterized by similar SERS intensities (the 10^−6^ M/10^−14^ M and 10^−8^ M/10^−12^ M concentrations). Therefore, to objectively estimate the lactoferrin concentration it is recommended to measure the SERS intensities of two or even more bands.

**Figure 11:**
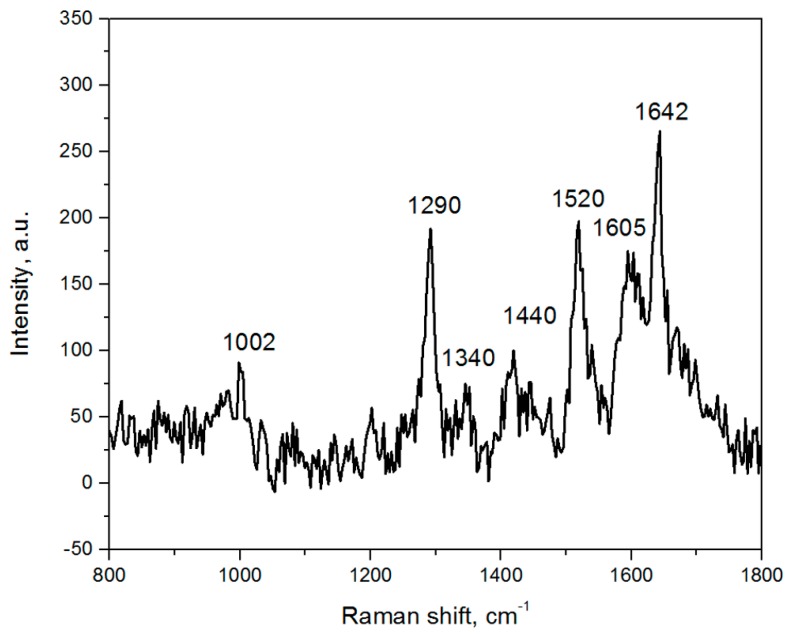
SERS spectrum of the lactoferrin molecules adsorbed on the silvered *por*-Si from 10^−18^ M solution and then protected with graphene.

To overcome the limitation of the 10^−16^ M lactoferrin concentration, at which it can be detected, we used the protection with graphene that was tested on the R6G molecules in the Section 3.3. In our experiments, we used the volume of 1 mL of the lactoferrin solutions. In case of the 10^−18^ M concentration, there are 10^−21^ moles of the protein molecules. Multiplication of this amount by Avogadro’s constant will give 602 lactoferrin molecules. The SERS-active area was 0.25 cm^2^ (=25 × 10^6^ μm^2^) so there are 25 × 10^4^ square maps of 100 μm^2^ area on it. Even if all the molecules are adsorbed on the SERS-active surface, the probability to catch one lactoferrin molecule in one map is negligibly small and can be calculated as 602/(25 × 10^4^) ≈ 0.24%. That is why we recorded several maps before the lactoferrin spectrum was found. It should be noted that the maps were chosen from the light areas of the sample surface, which contain 1–8 graphene layers. Figure 11 shows the SERS spectrum obtained for the lactoferrin molecules adsorbed from the solution of attomolar concentration on the silvered *por*-Si and then coated with graphene. We registered spectrum during 2 s. Despite the SERS spectrum intensity being relatively low, most of the lactoferrin Raman bands were presented there: 1002 cm^−1^ (Phe), 1290 cm^−1^ (Amide III), 1340 cm^−1^ (Trp), 1440 cm^−1^ (δCH_2_), 1605 cm^−1^ (Tyr) and 1642 cm^−1^ (Amide I).

Therefore, if the graphene-free substrates result in non-informative SERS spectra this can signify that the analyte concentration is lower than 10^−16^ M. To check the presence of the lactoferrin molecules the SERS-active substrate should be covered with the graphene-containing layer after immersion in the water solution. If the SERS mapping reveals the lactoferrin spectra, the concentration of the analyte will be 10^−18^ M.

Considering the non-uniform distribution of the lactoferrin molecules that have a tendency to get together on the substrates, it is reasonable to assume that a laser could hit the spot of several or even more molecules.

## 4. Conclusions

In the present work, we overcame the hurdles of the SERS-spectroscopy to study the biomolecules with high molecular weight using the lactoferrin as test analyte. To achieve the objectives of the work, the SERS-active substrates based on the silvered *por*-Si and the graphene protection of the analyte molecules were applied. We were able to measure the SERS spectra of the human lactoferrin molecules adsorbed on the silvered *por*-Si from the 10^−6^–10^−18^ M water solutions. It was found that the *por*-Si causes a negative surface potential of silver particles and their chemical resistivity to oxidation in contrast to those deposited on the *c*-Si surface. It must be caused by an extremely developed surface of the *por*-Si template enriched with silicon broken bonds. These features promoted the attraction of the positively charged lactoferrin molecules and prevented their interaction with metallic particles upon 473 nm laser excitation, which typically is not used to study proteins. The SERS spectra of lactoferrin adsorbed from 10^−6^ M solution were rather weak but the decrease of the concentration down to the 10^−10^ M level led to the enormous growth of the SERS signal. This effect was observed because at the 10^−6^–10^−8^ M concentrations protein molecules coalesced in accumulations of its nanoparticles that are too large for the uniform interaction with the electromagnetic field from the silver particles to provide an intensive SERS signal from the molecules. Lower concentrations led to the adsorption of much smaller particles (equal to monomers in size) that can be completely excited. Considering the tendency of lactoferrin oligomers breaking to the monomers while its concentration in solution decreases [59], this result can be used to reveal the structural form of lactoferrin. The SERS-spectra at the 10^−14^ and 10^−16^ M concentrations were less intensive and started to change (more prominent amorphous carbon bands) due to heating to the temperature of lactoferrin denaturation at about 350 K. To decrease the temperature in the laser spot to 340 K, the analyte molecules on the silvered *por*-Si were protected with graphene, which allowed detecting lactoferrin adsorbed from the 10^−18^ M solution. All the registered SERS spectra of lactoferrin contained most of the vibrational bands typical for this analyte. Therefore, the SERS-active substrates based on the silvered *por*-Si can be used for the label-free study of the correlation of vibrational wavenumbers of protein and to find lactoferrin in its water solutions at the concentrations down to attomolar. It should be mentioned that according to our knowledge a reliable SERS study of the lactoferrin molecules adsorbed on the solid SERS-active substrates has not been reported before. However, we have not yet estimated the lactoferrin concentration in any mixture of proteins or in the biological fluids. This research is now undergoing to apply the SERS-spectroscopy with the substrates based on the silvered *por*-Si both free and protected with graphene to detect lactoferrin in complex proteins’ solutions and physiological fluids.

## Figures and Tables

**Table 1:** Results of the EDX analysis.

Element	Series	At. %			
		Fresh Ag/*c*-Si	Aged Ag/*c*-Si	Fresh Ag/*por*-Si	Aged Ag/*por*-Si
C	K	18.32	20.71	16.74	20.70
O	K	2.03	3.92	4.25	4.30
Si	K	57.46	48.08	42.46	43.20
Ag	L	22.13	18.48	36.55	31.71

## Supplementary Materials

The following are available online at https://www.mdpi.com/2079-6374/9/1/34/s1: Figure S1: Original views of the SERS-active substrates: The samples of the silicon wafer with the square 0.5 × 0.5 cm area of the silvered por-Si placed in the Eppendorf tubes. Figure S2: SEM images of the silvered *por*-Si: (a) Top view; (b) cross-section, Figure S3: EDX spectrum typical for the silvered Si-based substrate, Figure S4: Electric field strength distribution simulated for the silvered *por*-Si: (a) Overview; (b) in the laser spot, Figure S5: (a,b) Temperature distributions and (c,d) isothermal contours simulated for the silvered *por*-Si: (a,c) free of graphene; (b,d) covered with graphene, Figure S6: Raman spectrum of lactoferrin (10^−4^ M water solution). Table S1: Parameters of the Raman and SERS-spectra of the graphene-containing films.
