# Supplementary material for: Surface Enhanced Raman Spectroscopy of Lactoferrin Adsorbed on Silvered Porous Silicon Covered with Graphene
Source: Biosensors (Basel). 2019 Feb 28;9(1):34. doi: 10.3390/bios9010034 (PMC6468514; doi:10.3390/bios9010034)
Supplement: Supplementary file 1 [file biosensors-09-00034-s001.pdf]

Supplementary Materials

Article

Surface Enhanced Raman Spectroscopy of Lactoferrin Adsorbed on Silvered Porous Silicon Covered with Graphene

Sergey Zavatski [1], Nadia Khinevich [1], Kseniya Girel [1], Sergey Redko [2], Nikolai Kovalchuk [3], Ivan Komissarov [3], Vladimir Lukashevich [4], Igor Semak [5], Kahramon Mamatkulov [6], Maria Vorobyeva [6], Grigory Arzumanyan [6,7] and Hanna Bandarenka [1,*]

1 Laboratory of Applied Plasmonics, Belarusian State University of Informatics and Radioelectronics, Minsk 220013, Belarus; sergeyzavatski13@gmail.com (S.Z.); khinevichnadia@gmail.com (N.K.); k.girel@bsuir.by (K.G.)
2 Laboratory of Materials and Structures of Nanoelectronics, Belarusian State University of Informatics and Radioelectronics, Minsk 220013, Belarus; ml.redkov@gmail.com
3 Laboratory of Integrated Micro- and Nanosystems, Belarusian State University of Informatics and Radioelectronics, Minsk 220013, Belarus; n.kovalchuk@bsuir.by (N.K.); komissarov@yahoo.com (I.K.)
4 Laboratory of Nutrition and Sports Physiology, Institute of Physiology of the National Academy of Sciences of Belarus, Minsk 220072, Belarus; lukashvs@rambler.ru
5 Department of Biochemistry, Belarusian State University, Minsk 220030, Belarus; semak@bsu.by
6 Laboratory of Neutron Physics, Joint Institute for Nuclear Research, Dubna 141980, Russia; hero170184@mail.ru (K.M.); vmu.chemist@mail.ru (M.V.); arzuman@jinr.ru (G.A.)
7 Dubna State University, Dubna 141982, Russia
* Correspondence: h.bandarenka@bsuir.by; Tel.: +37-517-293-8843

Received: 17 December 2018; Accepted: 20 February 2019; Published: 28 February 2019

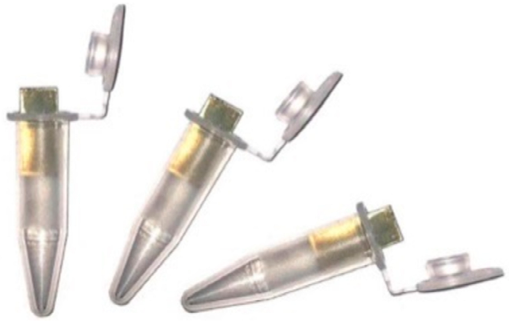

Figure S1. Original views of the SERS-active substrates: the samples of the silicon wafer with the square 0.5 × 0.5 cm area of the silvered *por*-Si placed in the Eppendorf tubes.

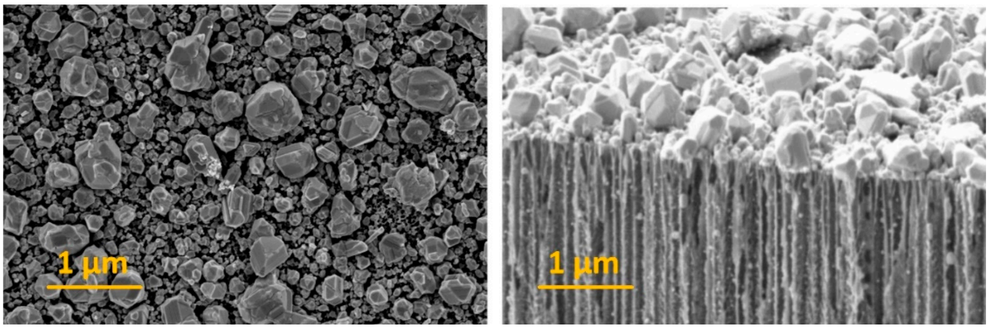

(**a**) (**b**)

Figure S2. SEM images of the silvered *por*-Si: (**a**) Top view; (b) Cross-section.

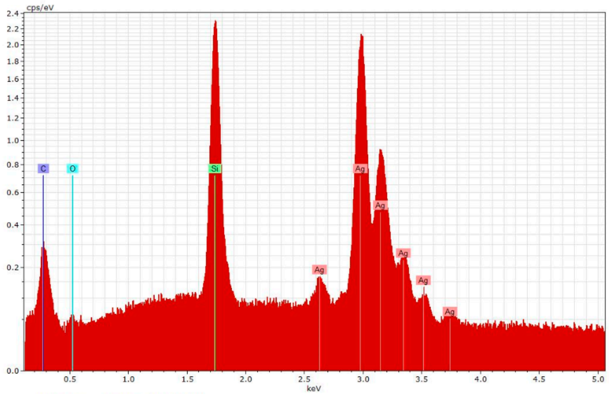

Figure S3. EDX spectrum typical for the silvered Si-based substrate.

Table S1. Parameters of the Raman and SERS-spectra of the graphene-containing films.

Spot	Spectrum	FWHM, cm^{-1}	Raman Shift, cm^{-1}		I_G/I_{2D}
			G band	2D band	
light	Raman, on *c*-Si	42	1587	2725	0.52
	SERS, on *por*-Si	29	1587	2725	0.39
dark	Raman, on *c*-Si	69	1582	2725	1.19
	SERS, on *por*-Si	84	1585	2723	2.86

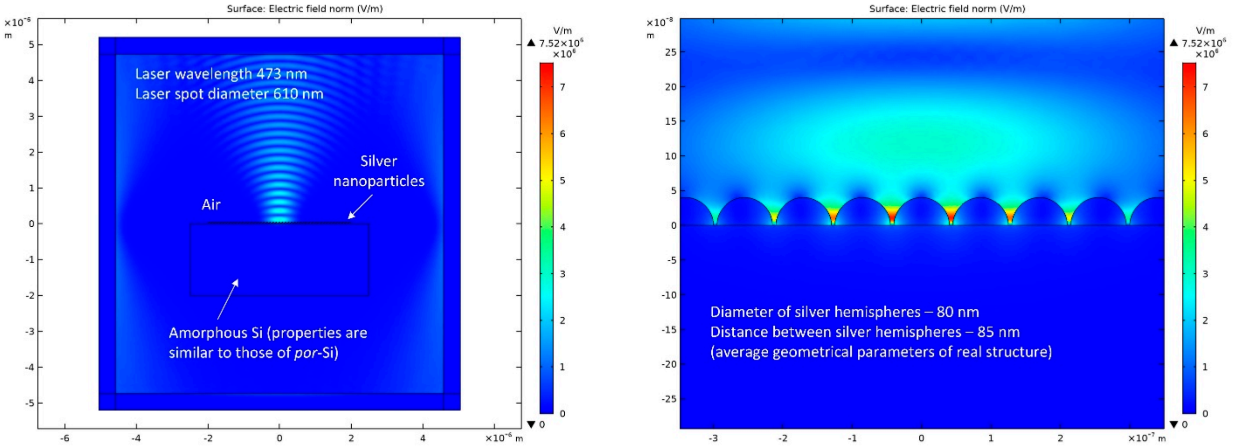

(**a**) (**b**)

Figure S4. Electric field strength distribution simulated for the silvered *por*-Si: (**a**) Overview; (**b**) In the laser spot.

The same electric field strength distribution was typical for the silvered *por*-Si covered with graphene.

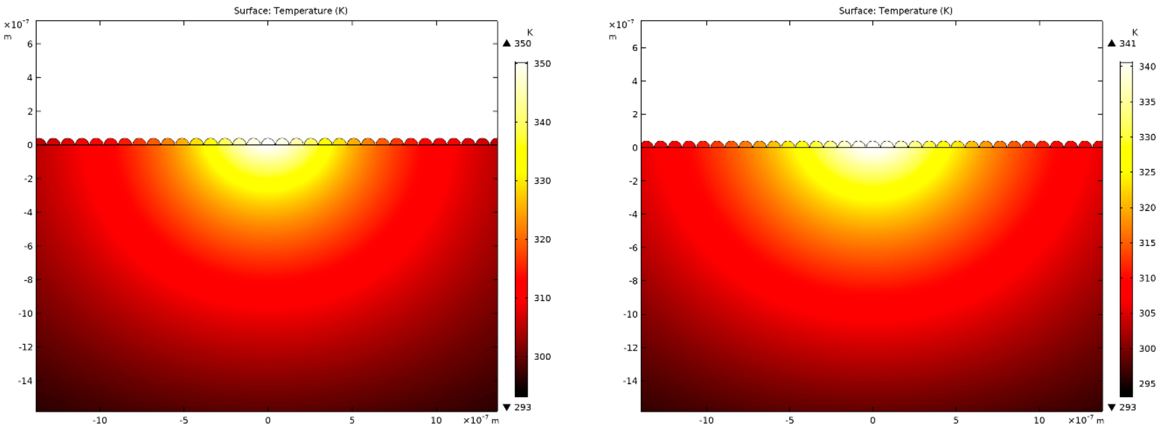

(**a**) (**b**)

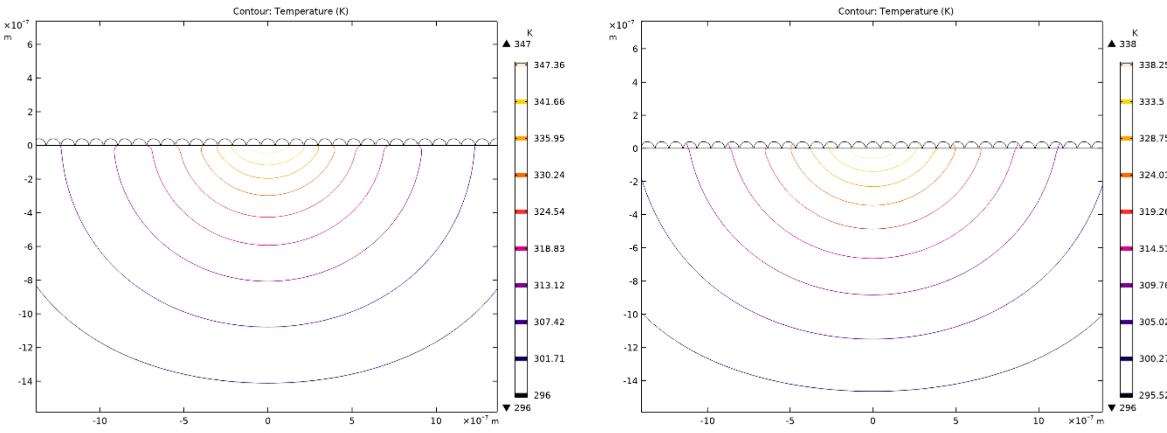

(**c**) (**d**)

Figure S5. (**a, b**) Temperature distributions and (**c, d**) isothermal contours simulated for the silvered *por*-Si: (**a, c**) free of graphene; (**b, d**) covered with graphene.

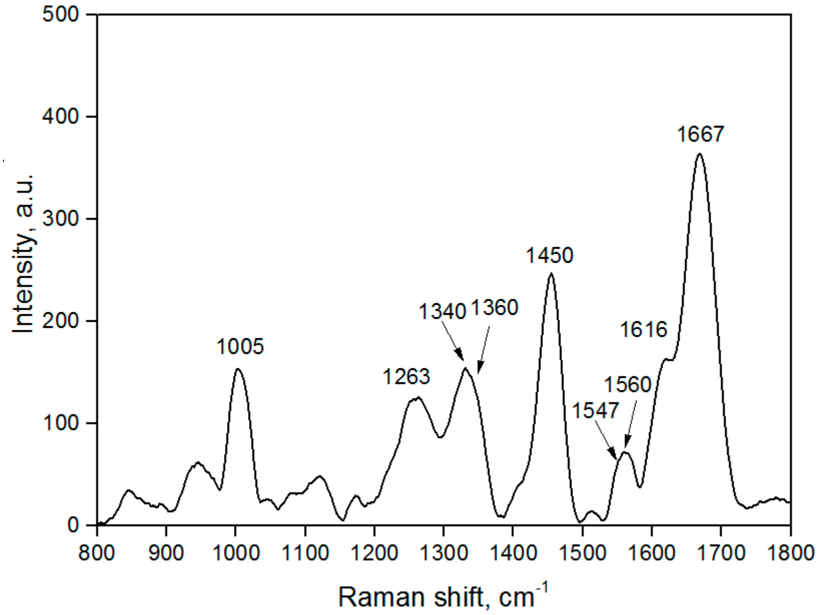

Figure S6. Raman spectrum of lactoferrin (10^{-4} M water solution).
